# Immune cell transcript modules reveal leukocyte heterogeneity in synovial biopsies of seronegative spondylarthropathy patients

**DOI:** 10.1186/1471-2474-15-446

**Published:** 2014-12-19

**Authors:** Robin M Hallett, Tracy Chew

**Affiliations:** Path Genomics, Hamilton, Ontario Canada; Department of Graduate Education and Research, Canadian Memorial Chiropractic College, 6100 Leslie Street, Toronto, Ontario M2H 3J1 Canada; Department of Pathology and Microbiology, Canadian Memorial Chiropractic College, 6100 Leslie Street, Toronto, Ontario M2H 3J1 Canada

**Keywords:** Spondylarthropathies, Ankylosing spondylitis, Immune infiltration, Inflammation, Rheumatology, Leukocyte, Transcriptional analysis, Gene signature, Metagene, Computational biology

## Abstract

**Background:**

The objective of this study was to identify leukocyte cell types found within the synovia of patients with seronegative spondylarthropathies (SpA), such as ankylosing spondylitis (AS), using transcription based analyses.

**Methods:**

Leukocyte transcriptional profiles obtained from the NCBI’s gene expression omnibus and prediction analysis of microarrays (PAM) was used to identify 25-gene leukocyte metagenes. Subsequently, transcriptional profiles from murine and clinical models of AS and SpA were interrogated to characterize the local infiltration of leukocytes in SpA synovia.

**Results:**

Analysis of a proteoglycan-induced murine model of AS reveals infiltration of dendritic cells, CD4+ T cells, monocytes, and natural killer cells to the spine. In human SpA and AS patients, transcriptional analysis of synovial biopsies revealed local infiltration of dendritic cells and CD4+ T cells.

**Conclusions:**

We identified leukocyte cell types that infiltrated the synovial of SpA patients. Our results imply a role for dendritic cells and CD4+ T cells in the local inflammation that underlies pathogenesis in patients with SpA.

**Electronic supplementary material:**

The online version of this article (doi:10.1186/1471-2474-15-446) contains supplementary material, which is available to authorized users.

## Background

Spondyloathropathies (SpA) represent a spectrum of inflammatory and degenerative joint diseases affecting the spine, with ankylosing spondylitis (AS) being among the most prevalent and debilitating worldwide. Primary manifestations of SpA involve axial manifestations such as sacroiliitis, and include both musculoskeletal and extra-articular symptoms. AS in particular is characterized by a variety of inflammatory conditions including joint and back pain, with severe cases progressing to spinal fusion and kyphosis leading to restricted mobility. While the disease is known to be inflammatory in nature, the specific mechanisms and cell types triggering inflammation within the joint are less well characterized.

Several genome-wide association studies and gene expression studies have been performed in AS and SpA patients, revealing several lines of evidence regarding the mechanisms of inflammation underlying disease [1–3]. Importantly, several important cellular processes have been implicated in the immune-mediated pathogenesis of AS, including antigen processing, the tumor necrosis factor alpha (TNFα)/nuclear factor kappa beta (NFκΒ)/interleukin 1 beta (IL-1β) axis, and the IL-23/IL-17/T helper 17 (Th17) axis. Serum TNFα and IL-6 levels correlate positively with disease [4], and TNFα inhibitors are the primary course of treatment for AS patients. In addition, the association of *IL12B* and *IL23R* genes in AS patients, as well as elevated serum and synovial levels of IL-17-related gene products suggests a role for the Th17/IL-23/IL-17 axis in pathology [5]. Importantly, these observations were derived from serological studies, and do not delineate local changes in a joint that may directly reflect important inflammatory processes underlying disease.

More importantly, it is unclear what inflammatory processes specifically occur within the synovia that are associated with joint/spinal inflammation, and which cell types are responsible for inflammatory cytokine production. To date, only a handful of studies have implicated a role for specific immune cell types involved in the inflammatory response in the joints of AS patients. The detection of FoxP3+ CD4+ T cells, IL-17-producting T cells, and KIR3DL2+ Th17 cells in the synovial fluid of patients with AS and with SpA implies a role for regulatory T cells in the local inflammatory processes in the joint [6–8]. Additionally, some early evidence of local infiltration of mast cells and phagocytic monocytes in the knee of AS patients by microscopy implies a role for these immune cell types in the local tissue [9]. However, this observation has not been validated by more comtemporary quanititative measures such as flow cytometry. Additional cells types associated with inflammation have been identified in the peripheral blood, but fail to delineate a relationship to the local inflammatory milieu [5, 10–13].

Importantly, gene expression profiles have been conducted from the synovial fluid of AS patients and from mouse models of AS. Given the availability of gene expression profiles from purified leukocyte populations as well as from the synovial biopsies of AS and SpA patients, we sought to identify the immune cell subtypes infiltrating the local inflammatory milieu of these patients.

## Methods

### Patients and samples

All data were publicly available and downloaded from the gene expression omnibus (GEO, http://www.ncbi.nlm.nih.gov/geo/). The leukocyte (GSE1133 & GSE22886), mouse AS (GSE41039) and patient biopsy (GSE41038) datasets were downloaded and processed independently [14–17]. For Affymetrix data, the raw .CEL files comprising each dataset were download and normalized using the Robust Multichip Algorithm (RMA) to generate probe set intensities [18]. For Illumina data, normalized data was downloaded directly from GEO. All human data were downloaded from a publicly available database, and were previously published. No new patients or samples were collected for this study.

### Prediction analysis of microarrays (PAM)

PAM was downloaded as an excel add-in from http://www-stat.stanford.edu/~tibs/PAM/, and was used according to the available manual. Briefly, we used this algorithm to interrogate the human leukocyte series for the top 25 class genes where each different population of purified leukocytes was an independent class. Leukocyte class assignment was made as follows: Monocytes (GSM18871 & 18872), DCs (GSM18873 & 18874), NK cells (GSM18875 & 18876), CD4+ T cells (GSM18877 & 18878), CD8+ T cells (GSM18879 & 18880), and B cells (GSE18881 & 18882).

### Evaluation of leukocyte metagenes

For cross-platform comparisons, datasets were collapsed to single genes based on highest mean average and mapped based on Unigene ID. To evaluate leukocyte metagenes, the expression values for each gene was transformed such that the mean and standard deviation were set to 0 and 1 in each dataset, respectively. A metagene score was calculated for each sample as follows:

$$\frac{\sum_{i\in p}x_{i}}{n_{p}}$$

where *x* is the transformed expression, *n* is the number of genes in class set P and P is the set of class marker genes for a particular metagene [19–21].

### Statistical analysis

T-tests were used to compare indices between control and spondylarthropathy samples in both murine and human datasets. All tests were two-sided and a p-value of 0.05 or less was considered statistically significant.

## Results

### Identification of leukocyte expression metagenes

**Figure 1:**
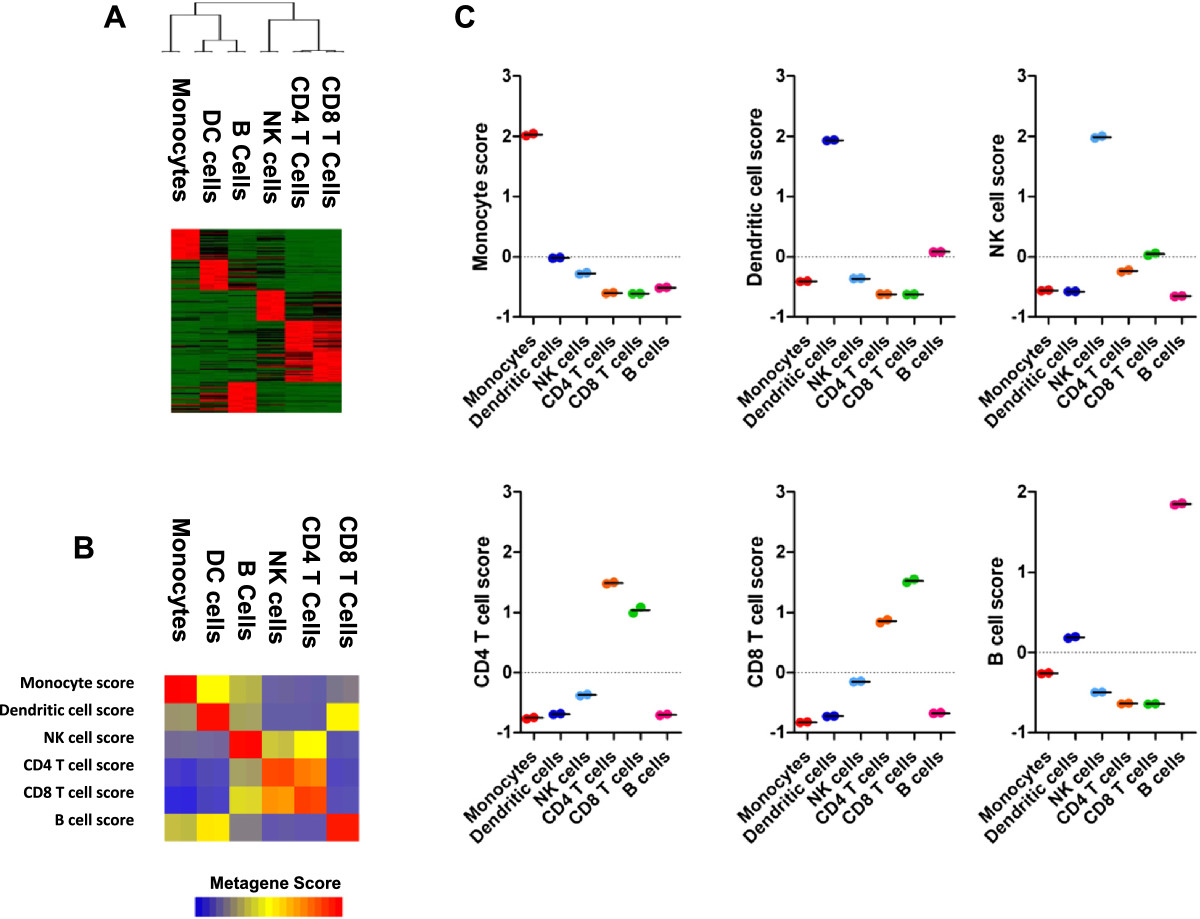
**Gene expression measurements uniquely identify immune cell subsets** ***.*** **(A)** Expression of top 25 specific immune cell transcripts among various preparations of purified immune cells. **(B)** Heatmap representation of immune metagene scores calculated from the top 25 specific immune cell transcripts for each cell type. **(C)** Scatter plot of subtype-specific metagene scores for each purified immune cell subtype.

Whereas the underlying mechanisms behind AS and other SpA are known to be inflammatory in nature, the specific leukocyte cell populations that drive inflammation are unknown. Hence, we sought to measure infiltration of specific leukocyte cell populations within synovial biopsies of experimental and clinical models of AS and SpA. To this end, we identified leukocyte transcript modules, which could be implemented to measure leukocyte infiltration in patient biopsies for which the global gene expression profile is known. Based on publicly available global gene expression profiling data of the human transcriptome, we identified samples representing multiple purified leukocyte cell populations including monocytes, dendritic cells (DC), B cells, natural killer (NK) cells, CD4+ T cells and CD8+ T cells. We used PAM [22] to identify a gene expression based classifier that could discriminate between leukocyte cell type according to patterns in gene expression. Briefly, PAM is an efficient tool to identify molecular patterns that is applicable to gene expression data, and uses nearest shrunken centroids to identify subsets of genes that best characterize sample classes. We used PAM to identify a 150 gene classifier that comprised the top 25 marker genes associated with each leukocyte cell type (see Additional file 1: Table S1). This composite gene signature is known as a metagene, and gene clusters representing each leukocyte metagene is shown in Figure 1A. To test whether the expression of each leukocyte metagene was highly discriminatory between specific populations of leukocytes, we calculated immune cell metagene scores for each cell type across all cell types (Figures 1B and C). For example, the average monocyte metagene score was highest in the purified monocyte cell population, and negative in each other leukocyte population. Although we observed some cross-specificity between the CD4 and CD8 T cell signatures, the leukocyte metagene score was highest in the target cell population for each leukocyte cell type, supporting the overall validity of the approach. To further validate this approach we downloaded additional independent gene expression profiles from purified leukocyte populations and tested the capacity of the various metagenes to accurately discriminate between the cell types [17]. Importantly, the metagene signatures were sufficient to stratify these various leukocyte cell types (see Additional file 2: Figure S1). Based on these data, we concluded that leukocyte metagene signatures could be implemented to measure infiltration of specific leukocyte cell populations in patient biopsies.

### Leukocyte metagenes identify local infiltration of multiple leukocyte subsets in a murine model of AS

**Figure 2:**
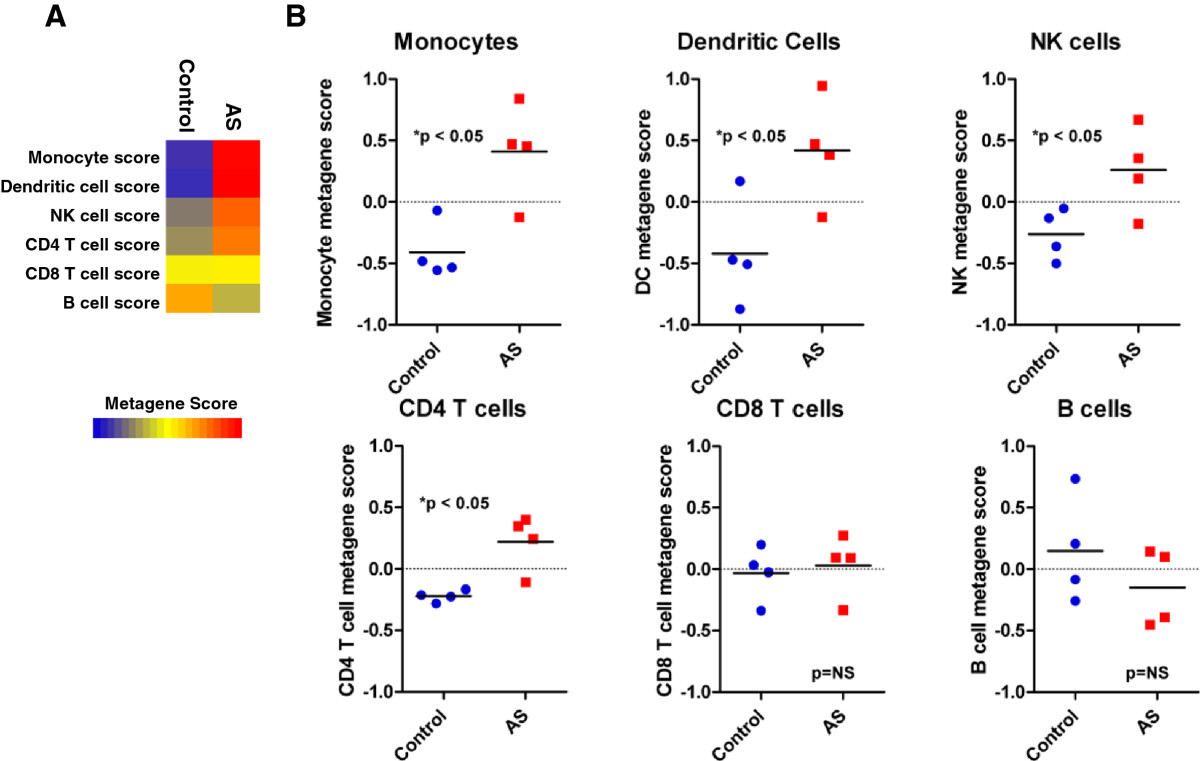
**Synovial biopsies of AS mice contain increased numbers of monocytes, dendritic cells, NK cells, and CD4+ T cells. (A)** Heatmap representation of immune metagene scores from synovial biopsies of control mice or peptidoglycan-induced AS mice. **(B)** Scatter plot of subtype-specific metagene scores from synovial biopsies of control or AS mice. *indicates statistical significance between AS biopsies and control for a given leukocyte subset by student’s T-test.

Although the overarching objective of this work was to was identify populations of leukocytes that might mediate inflammation in human patients with either AS or SpA, we first sought to examine the expression of leukocyte metagenes in a mouse model of AS. In this case, the mouse model offers several advantages over the human samples, including samples being harvested from mice with identical genetic backgrounds, as well as whole spine samples being obtained for global gene expression profiles. Briefly, we downloaded publicly available gene expression profiles of control (n = 4) and experimental (n = 4) mouse spines based on the proteoglycan-induced AS model in BALB/c mice [23]. We observed expression differences between control and experimental mice for several of the leukocyte metagenes (Figure 2A). Specifically, we observed increased monocyte, DC, NK, and CD4+ T cell metagene expression within the spines of mice induced with experimental AS (Figure 2B). Hence, we conclude that in an experimental mouse model of AS, the disease is associated with increased monocyte, DC, NK cells, and CD4+ T cell infiltration relative to control animals.

### Leukocyte metagenes identify differential leukocyte infiltration in synovial biopsies of patients with seronegative SpA

**Figure 3:**
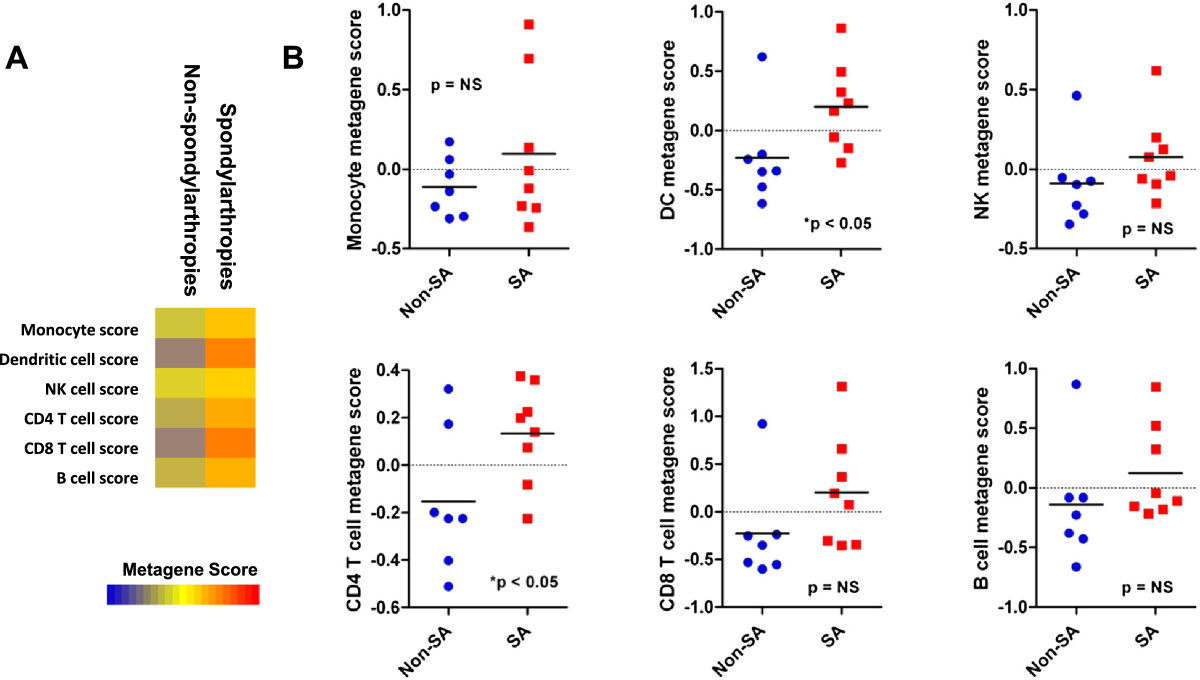
**Synovial biopsies of AS and seronegative spondylarthropathy (SpA) patients contain increased numbers of dendritic cells and CD4+ T cells compared to control patients. (A)** Heatmap representation of immune metagene scores from synovial biopsies of control donors or AS/SpA patients. **(B)** Scatter plot of subtype-specific metagene scores from synovial biopsies of control donors or AS/SpA patients. *indicates statistical significance between AS biopsies and control for a given leukocyte subset by student’s T-test.

To extend these findings in human patients, we examined the expression of leukocyte metagenes in synovial biopsies extracted from patients with seronegative SpA or in control biopsies. Briefly, we downloaded publicly available gene expression profiling data for the samples described above, and compared leukocyte metagene expression in biopsies derived from the 8 SpA patients (2 AS, 6 SpA) with 7 control biopsies (4 normal, 3 OA). Similar to the mouse model data, we observed differences in leukocyte metagene expression among the control and SpA patient biopsies (Figure 3A). Specifically, we observed increased DC and CD4+ T cell metagene expression in the SpA biopsies relative to control biopsies (Figure 3B). Interestingly, we did not observe increased expression of monocyte and NK cell metagene expression in the human SpA biopsies relative to control, whereas these metagenes were significantly more highly expressed in experimental mouse biopsies. A post-hoc analysis of individual genes comprising the differentially expressed leukocyte metagenes, revealed that metagenes were upregulated as a whole, and the observed increases were not driven by dramatic up-regulation relatively few genes (see Additional file 3: Figure S2). Importantly, this suggests that our observations are not confounded by dramatic changes in single genes, and likely represent true measurements of infiltration.

Taken together with our previous data, these findings strongly suggest that the joints of patients with AS/SpA contain increased leukocyte infiltrate that primarily comprises DC and CD4+ T cells relative to control patient joints.

## Discussion

This study identifies infiltrating leukocyte subtypes within the local tissue of patients suffering from seronegative SpA. To our knowledge, this is the first study to relate leukocyte cell types found in peripheral blood to their infiltration within the local inflammatory milieu of the joints in these patients. Previous studies identified increased numbers of IL-17- and IL-22-producing CD4+ T cells [5, 10], but their specific role in the pathogenesis of disease remains unclear. Furthermore, their presence within the synovial of inflamed joints in the spine has not been elucidated. As gene expression profiles of specific T cell subsets, including Th17, γ/δ, and regulatory T cells, is not available, this study is not able to distinguish these specific T cell subsets. An interrogation of these cell types in the local tissue would be an important follow-up study, as a number of T cell subsets have been implicated in disease from peripheral blood analyses.

Previous studies also identified various biomarkers in the peripheral blood of AS patients that correlate with disease. For example, Bal *et al.*[4] demonstrate that AS patients have increased circulating levels of TNFα; interestingly, monocyte-derived DCs and macrophages are an important source of TNFα and play a crucial role in the initiation of a local inflammatory response. Additionally, several TNFα-related genes have been identified in the positively correlated immune cell subsets, implying an important role for this pathway in immune pathogenesis of disease. Although we did not observe significantly increased expression of monocyte markers in SpA patients, a proportion of the patients in this sample set did exhibit increased monocyte metagene expression. Whether this trend would be statistically significant in a larger sample size is unclear. Together with the observation that monocytes were found at increased levels in a mouse model of AS, it is clear that infiltrating monocytic cells including monocyte-derived DCs are associated with the disease state. Similarly, we have shown here that professional antigen presenting cells including monocyte-derived cells and DCs are upregulated in AS synovial, as previously observed in peripheral blood. Interestingly, DCs have been shown to be a source of elevated serum ERAP1 expression in AS patients, implying a dysfunctional role of DCs in antigen presentation leading to inflammation [24]. Finally, the IL-23/Th17/IL-17 axis has been implicatied in AS pathogenesis [1–3]. Here, we report increased levels of CD4+ T cells in within the synovial fluid of AS/SpA patients. Whether these cells also express IL-17, or include Th17 cells, is not clear, as transcriptional profiles of purified Th17 cells are not available for meta-analysis of this type.

Importantly, immune pathways previously identified to underlie AS pathogenesis have not been identified in the local environment in our analysis. For example, Baek *et al.* observed increased levels of IL-4+ CD8+ T cells in the peripheral blood of AS patients [10]. In our study, we do not observe CD8+ T cells levels within the synovia, implying that this cell type may not play as important a role in the local tissue as it does in the periphery. Additionally, a recent identification of abzymes (antibodies with catalytic activity) has been correlated to SpA [25], despite the absence of B cells within the local tissue. Indeed, extra-articular symptoms of AS and other SpA may involve such cell types; whether these cell types infiltrate the local environment of an extra-articular site of inflammation is an important question and remains unclear.

## Conclusions

Our data suggest that local inflammatory processes underlying SpA cannot be deduced based on biomarkers identified in peripheral blood. While peripheral blood markers serve as important diagnostic tools, the local environment is responsible to eliciting inflammation within the joint and should be the basis for pharmacological interventions. Therefore, a closer investigation of the local inflammatory responses underlying a variety of inflammatory arthritic diseases, including AS/SpA, is an important first step in understanding and targeting pathways involved in pathogenesis of disease.

## Electronic supplementary material

Additional file 1: Table S1: Leukocyte metagene composition. Top 25 genes comprising the leukocyte gene signature for monocytes, dendritic cells, natural killer cells, CD4+ T cells, CD8+ T cells, and B cells. (PDF 58 KB)

Additional file 2: Figure S1: Validation of immune cell metagenes using independent data. Metagene scores for immune cell subtypes using an independent gene expression dataset. (PDF 274 KB)

Additional file 3: Figure S2: Gene-centric evaluation of differentially expressed leukocyte metagenes in the mouse (A-D) and human (E & F) datasets. Description of data: Heatmap representation of gene expression in cell types identified in this study. Genes comprising the leukocyte metagene were analyzed for differential expression between healthy and diseases, and indicate no clear pattern of single gene-driven differences in metagene scores. (PDF 61 KB)

Below are the links to the authors’ original submitted files for images.

Authors’ original file for figure 1

Authors’ original file for figure 2

Authors’ original file for figure 3
