# Supplementary material for: Immune cell transcript modules reveal leukocyte heterogeneity in synovial biopsies of seronegative spondylarthropathy patients
Source: BMC Musculoskelet Disord. 2014 Dec 19;15:446. doi: 10.1186/1471-2474-15-446 (PMC4320502; doi:10.1186/1471-2474-15-446)
Supplement: Supplementary file 1 — Additional file 1: Table S1: Leukocyte metagene composition. Top 25 genes comprising the leukocyte gene signature for monocytes, dendritic cells, natural killer cells, CD4+ T cells, CD8+ T cells, and B cells. (PDF 58 KB) [file 12891_2014_2396_MOESM1_ESM.pdf]

Cell type	Gene	Description
Monocyte	FPR1	formyl peptide receptor 1 /// formyl peptide receptor 1
	CSTA	cystatin A (stefin A)
	MNDA	myeloid cell nuclear differentiation antigen /// myeloid cell nuclear differentiation antigen
	RNF130	ring finger protein 130
	S100A8	S100 calcium binding protein A8 (calgranulin A)
	IFI30	interferon, gamma-inducible protein 30
	MEGF9	multiple EGF-like-domains 9
	LST1	leukocyte specific transcript 1
	CEBPB	CCAAT/enhancer binding protein (C/EBP), beta
	SLC7A7	solute carrier family 7 (cationic amino acid transporter, y+ system), member 7
	PPT1	palmitoyl-protein thioesterase 1 (ceroid-lipofuscinosis, neuronal 1, infantile)
	CRISPLD2	cysteine-rich secretory protein LCCL domain containing 2
	TKT	transketolase (Wernicke-Korsakoff syndrome)
	FLJ22662	hypothetical protein FLJ22662
	CD1D	CD1d molecule /// CD1d molecule
	LGALS1	lectin, galactoside-binding, soluble, 1 (galectin 1)
	CD163	CD163 molecule
	RAB31	RAB31, member RAS oncogene family
	HSPA1A	heat shock 70kDa protein 1A
	LILRA2	leukocyte immunoglobulin-like receptor, subfamily A (with TM domain), member 2
	FGL2	fibrinogen-like 2
	CTSB	cathepsin B
	SEPX1	selenoprotein X, 1
	MYD88	myeloid differentiation primary response gene (88)
	LMO2	LIM domain only 2 (rhombotin-like 1)
Dencritic Cell	LOC651629	similar to Ig kappa chain V-I region Walker precursor
	ITM2C	integral membrane protein 2C /// integral membrane protein 2C
	IL1B	interleukin 1, beta
	ALOX5AP	arachidonate 5-lipoxygenase-activating protein
	IGKC	Immunoglobulin kappa constant
	GAS6	growth arrest-specific 6
	CSF2RB	colony stimulating factor 2 receptor, beta, low-affinity (granulocyte-macrophage) /// colony stimulating factor 2 receptor, beta, low-affinity (granulocyte-macrophage)
	TXNDC5	thioredoxin domain containing 5 /// thioredoxin domain containing 5
	PACAP	proapoptotic caspase adaptor protein
	IGKC /// IGKV1-5	immunoglobulin kappa constant /// immunoglobulin kappa variable 1-5
	HYOU1	hypoxia up-regulated 1
	IGHA1 /// IGHA2	immunoglobulin heavy constant alpha 1 /// immunoglobulin heavy constant alpha 2 (A2m marker)
	LEPREL1	leprecan-like 1
	HLA-DQA1 /// HLA-DQA2 /// LOC650946	major histocompatibility complex, class II, DQ alpha 1 /// major histocompatibility complex, class II, DQ alpha 2 /// similar to HLA class II histocompatibility antigen, DQ(1) alpha chain precursor (DC-4 alpha chain)
	IGH@ /// IGHG1 /// IGHG2 /// IGHG3 /// IGHM /// LOC652848	immunoglobulin heavy locus /// immunoglobulin heavy constant gamma 1 (G1m marker) /// immunoglobulin heavy constant gamma 2 (G2m marker) /// immunoglobulin heavy constant gamma 3 (G3m marker) /// immunoglobulin heavy constant mu /// anti-RhD monoclonal T125 gamma1 heavy chain
	CUEDC1	CUE domain containing 1
	C20ORF103	chromosome 20 open reading frame 103
	TNFRSF21	tumor necrosis factor receptor superfamily, member 21
	MAN2B1	mannosidase, alpha, class 2B, member 1
	BLNK	B-cell linker
	HLA-DRB4	major histocompatibility complex, class II, DR beta 4
	IRF4	interferon regulatory factor 4
	IGLC1	immunoglobulin lambda constant 1 (Mcg marker)
	HLA-DPA1	major histocompatibility complex, class II, DP alpha 1
	LOC652745	similar to Ig kappa chain V-I region Walker precursor

Cell type	Gene	Description
NK Cell	MYOM2	myomesin (M-protein) 2, 165kDa /// myomesin (M-protein) 2, 165kDa
	GZMH	granzyme H (cathepsin G-like 2, protein h-CCPX) /// granzyme H (cathepsin G-like 2, protein h-CCPX)
	ARL4C	ADP-ribosylation factor-like 4C
	GNLY	granulysin
	HOP	homeodomain-only protein /// homeodomain-only protein
	KLRD1	killer cell lectin-like receptor subfamily D, member 1
	PRKCH	protein kinase C, eta
	FCGR3B	Fc fragment of IgG, low affinity IIIb, receptor (CD16b)
	FLJ20699	hypothetical protein FLJ20699
	ID2	inhibitor of DNA binding 2, dominant negative helix-loop-helix protein
	IL18RAP	interleukin 18 receptor accessory protein
	IFITM1	interferon induced transmembrane protein 1 (9-27)
	CCL5	chemokine (C-C motif) ligand 5 /// chemokine (C-C motif) ligand 5
	CD160	CD160 molecule
	RUNX3	runt-related transcription factor 3
	CST7	cystatin F (leukocystatin)
	TGFBR3	transforming growth factor, beta receptor III (betaglycan, 300kDa)
	MBP	myelin basic protein
	TPST2	tyrosylprotein sulfotransferase 2
	GZMA	granzyme A (granzyme 1, cytotoxic T-lymphocyte-associated serine esterase 3) /// granzyme A (granzyme 1, cytotoxic T-lymphocyte-associated serine esterase 3)
	FAM108A1	family with sequence similarity 108, member A1 /// family with sequence similarity 108, member A1
	NCAM1	neural cell adhesion molecule 1
	STOM	stomatin
	FYN	FYN oncogene related to SRC, FGR, YES
	RGS3	regulator of G-protein signalling 3
CD4 T-cell	TRAC	T cell receptor alpha constant /// T cell receptor alpha constant
	IL7R	interleukin 7 receptor /// interleukin 7 receptor
	CD3D	CD3d molecule, delta (CD3-TCR complex)
	CD6	CD6 molecule /// CD6 molecule
	DGKA	diacylglycerol kinase, alpha 80kDa
	TRA@ /// TRAC	T cell receptor alpha locus /// T cell receptor alpha locus /// T cell receptor alpha constant /// T cell receptor alpha constant
	DDX17	DEAD (Asp-Glu-Ala-Asp) box polypeptide 17
	SATB1	special AT-rich sequence binding protein 1 (binds to nuclear matrix/scaffold-associating DNA's)
	MGC17330	HGFL gene /// HGFL gene
	PLSCR3	phospholipid scramblase 3
	GOLGA8B	golgi autoantigen, golgin subfamily a, 8B
	TRBV21-1 /// TRBV19 /// TRBV5-4 /// TRBV3-1 /// TRBC1 /// LOC647353	T cell receptor beta variable 21-1 /// T cell receptor beta variable 19 /// T cell receptor beta variable 5-4 /// T cell receptor beta variable 3-1 /// T cell receptor beta constant 1 /// similar to T-cell receptor beta chain V region CTL-L17 precursor
	BCL11B	B-cell CLL/lymphoma 11B (zinc finger protein)
	TRBV19 /// TRBC1	T cell receptor beta variable 19 /// T cell receptor beta variable 19 /// T cell receptor beta constant 1 /// T cell receptor beta constant 1
	MAL	mal, T-cell differentiation protein
	LAT	linker for activation of T cells
	JARID1D	jumonji, AT rich interactive domain 1D
	TNFRSF25	tumor necrosis factor receptor superfamily, member 25
	TRA@ /// TRDV2 /// TRAV20 /// TRAC	T cell receptor alpha locus /// T cell receptor delta variable 2 /// T cell receptor alpha variable 20 /// T cell receptor alpha constant
	DNAJB1	DnaJ (Hsp40) homolog, subfamily B, member 1
	RPS4Y1	ribosomal protein S4, Y-linked 1
	IL6ST	Interleukin 6 signal transducer (gp130, oncostatin M receptor)
	C16ORF30	chromosome 16 open reading frame 30
	RPS3	ribosomal protein S3
	PLA2G4B	phospholipase A2, group IVB (cytosolic)

Cell type	Gene	Description
CD8 T-cell	GZMK	granzyme K (granzyme 3; tryptase II) /// granzyme K (granzyme 3; tryptase II)
	KNS2	Kinesin 2
	SNRK	SNF related kinase
	KLRK1	killer cell lectin-like receptor subfamily K, member 1
	ATHL1	ATH1, acid trehalase-like 1 (yeast)
	ATM	ataxia telangiectasia mutated (includes complementation groups A, C and D)
	CD8A	CD8a molecule /// CD8a molecule
	CCR7	chemokine (C-C motif) receptor 7 /// chemokine (C-C motif) receptor 7
	GOLGA8A	golgi autoantigen, golgin subfamily a, 8A
	ITK	IL2-inducible T-cell kinase
	OGT	O-linked N-acetylglucosamine (GlcNAc) transferase (UDP-N-acetylglucosamine:polypeptide-N-acetylglucosaminyl transferase)
	TRGC2 /// TRGV2 /// TRGV9 /// TARP /// LOC642083	T cell receptor gamma constant 2 /// T cell receptor gamma variable 2 /// T cell receptor gamma variable 9 /// TCR gamma alternate reading frame protein /// hypothetical protein LOC642083
	DUSP2	dual specificity phosphatase 2
	NELL2	NEL-like 2 (chicken) /// NEL-like 2 (chicken)
	XIST	X (inactive)-specific transcript
	TRAF3IP3	TRAF3 interacting protein 3
	HIST1H2BG	histone 1, H2bg
	GIMAP5	GTPase, IMAP family member 5
	KLRG1	killer cell lectin-like receptor subfamily G, member 1
	LCK	lymphocyte-specific protein tyrosine kinase
	LEF1	lymphoid enhancer-binding factor 1
	TCF7	transcription factor 7 (T-cell specific, HMG-box)
	CD2	CD2 molecule /// CD2 molecule
	SPOCK2	sparc/osteonectin, cwcv and kazal-like domains proteoglycan (testican) 2
	PASK	PAS domain containing serine/threonine kinase
B-cell	POU2AF1	POU domain, class 2, associating factor 1
	VPREB3	pre-B lymphocyte gene 3
	P2RX5	purinergic receptor P2X, ligand-gated ion channel, 5
	CD24	CD24 molecule
	HLA-DMB	major histocompatibility complex, class II, DM beta /// major histocompatibility complex, class II, DM beta
	CD79A	CD79a molecule, immunoglobulin-associated alpha /// CD79a molecule, immunoglobulin-associated alpha
	FCER2	Fc fragment of IgE, low affinity II, receptor for (CD23)
	QRSL1	glutaminyl-tRNA synthase (glutamine-hydrolyzing)-like 1
	CD22 /// MAG	CD22 molecule /// myelin associated glycoprotein
	HLA-DQB1	major histocompatibility complex, class II, DQ beta 1 /// major histocompatibility complex, class II, DQ beta 1
	ODC1	ornithine decarboxylase 1
	AKAP2 /// PALM2-AKAP2	A kinase (PRKA) anchor protein 2 /// PALM2-AKAP2 protein
	TCL1A	T-cell leukemia/lymphoma 1A
	MS4A1	membrane-spanning 4-domains, subfamily A, member 1
	HLA-DRA	major histocompatibility complex, class II, DR alpha
	KIAA0746	KIAA0746 protein
	SWAP70	SWAP-70 protein
	CD19	CD19 molecule
	LYN	v-yes-1 Yamaguchi sarcoma viral related oncogene homolog
	CD37	CD37 molecule
	CD72	CD72 molecule
	HLA-DQB1 /// LOC650557	major histocompatibility complex, class II, DQ beta 1 /// major histocompatibility complex, class II, DQ beta 1 /// similar to HLA class II histocompatibility antigen, DQ(W1.1) beta chain precursor (DQB1*0501) /// similar to HLA class II histocompatibility antigen, DQ(W1.1) beta chain precursor (DQB1*0501)
	HLA-DMA	major histocompatibility complex, class II, DM alpha
	BRDG1	BCR downstream signaling 1
	CHPT1	choline phosphotransferase 1
