# Supplementary figures and images for: Immune cell transcript modules reveal leukocyte heterogeneity in synovial biopsies of seronegative spondylarthropathy patients
Source: BMC Musculoskelet Disord. 2014 Dec 19;15:446. doi: 10.1186/1471-2474-15-446 (PMC4320502; doi:10.1186/1471-2474-15-446)

Supplementary Figure 1. Validation of immune cell metagenes using independent data

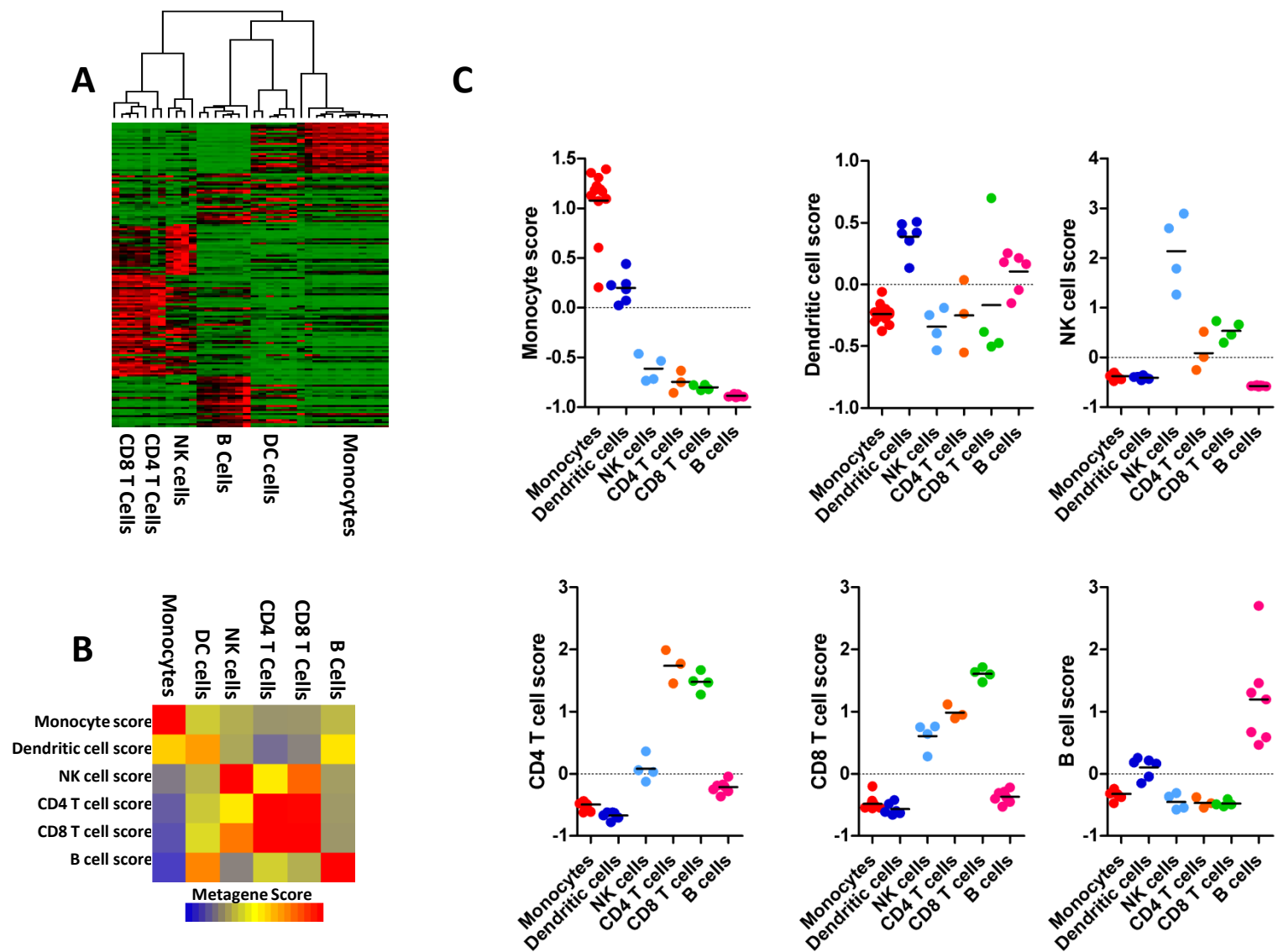

Supplement: Supplementary file 2 — Additional file 2: Figure S1: Validation of immune cell metagenes using independent data. Metagene scores for immune cell subtypes using an independent gene expression dataset. (PDF 274 KB) [file 12891_2014_2396_MOESM2_ESM.pdf]

**A**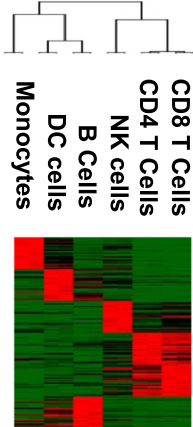**B**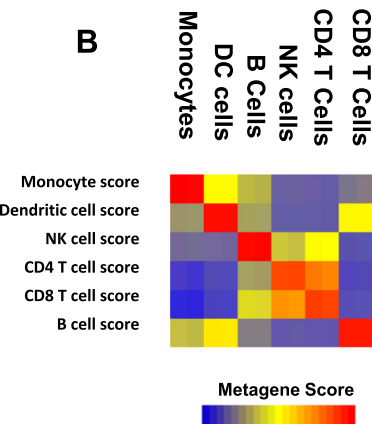**C**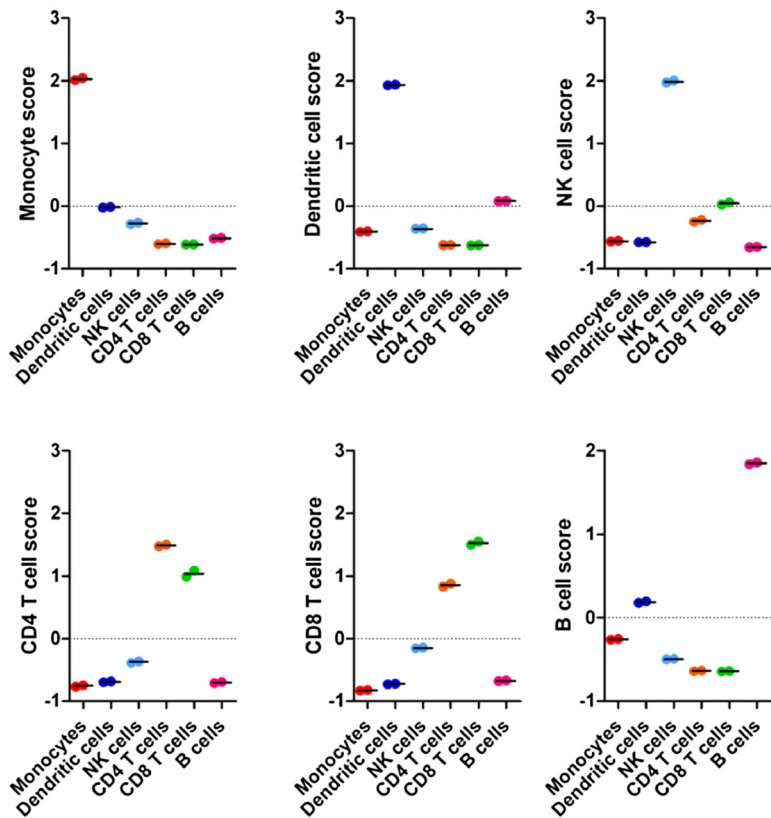

Supplement: Supplementary file 4 — Authors’ original file for figure 1 [file 12891_2014_2396_MOESM4_ESM.pdf]

**A**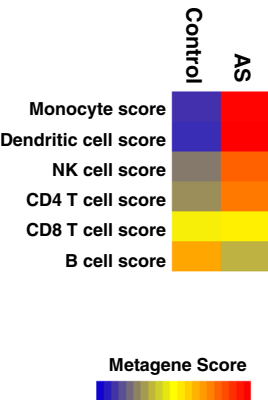**B**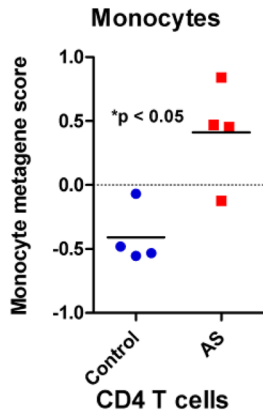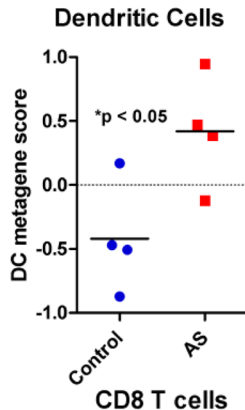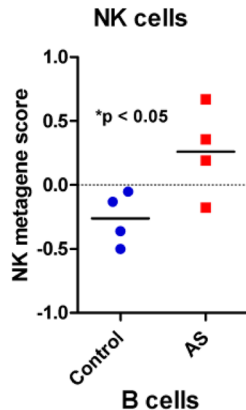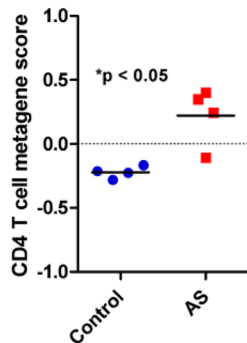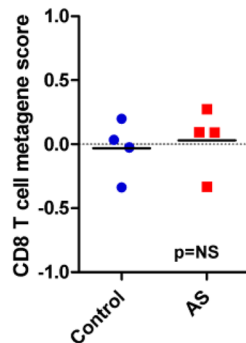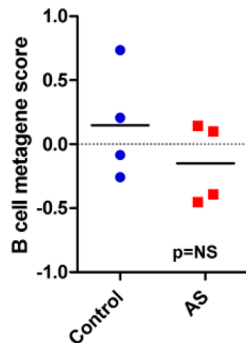

Supplement: Supplementary file 5 — Authors’ original file for figure 2 [file 12891_2014_2396_MOESM5_ESM.pdf]

**A**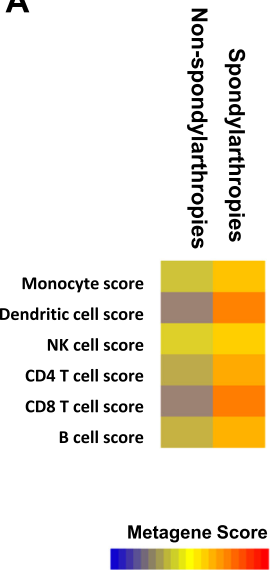**B**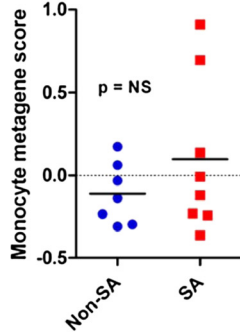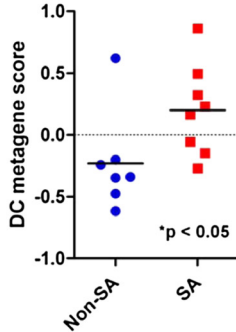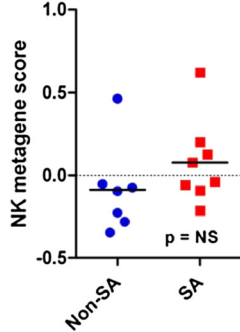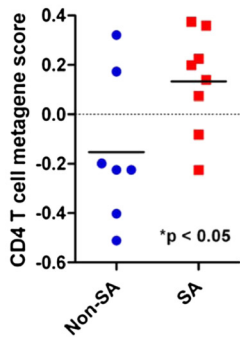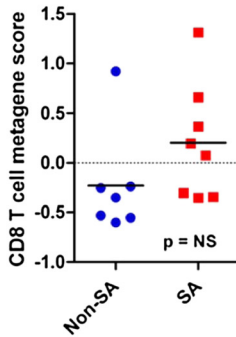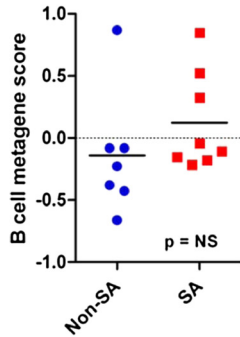

Supplement: Supplementary file 6 — Authors’ original file for figure 3 [file 12891_2014_2396_MOESM6_ESM.pdf]
