# Supplementary material for: Immune cell transcript modules reveal leukocyte heterogeneity in synovial biopsies of seronegative spondylarthropathy patients
Source: BMC Musculoskelet Disord. 2014 Dec 19;15:446. doi: 10.1186/1471-2474-15-446 (PMC4320502; doi:10.1186/1471-2474-15-446)

Supplementary Figure 2. Gene-centric evaluation of differentially expressed leukocyte metagenes in the mouse **(A-D)** and human datasets **(E&F)**.

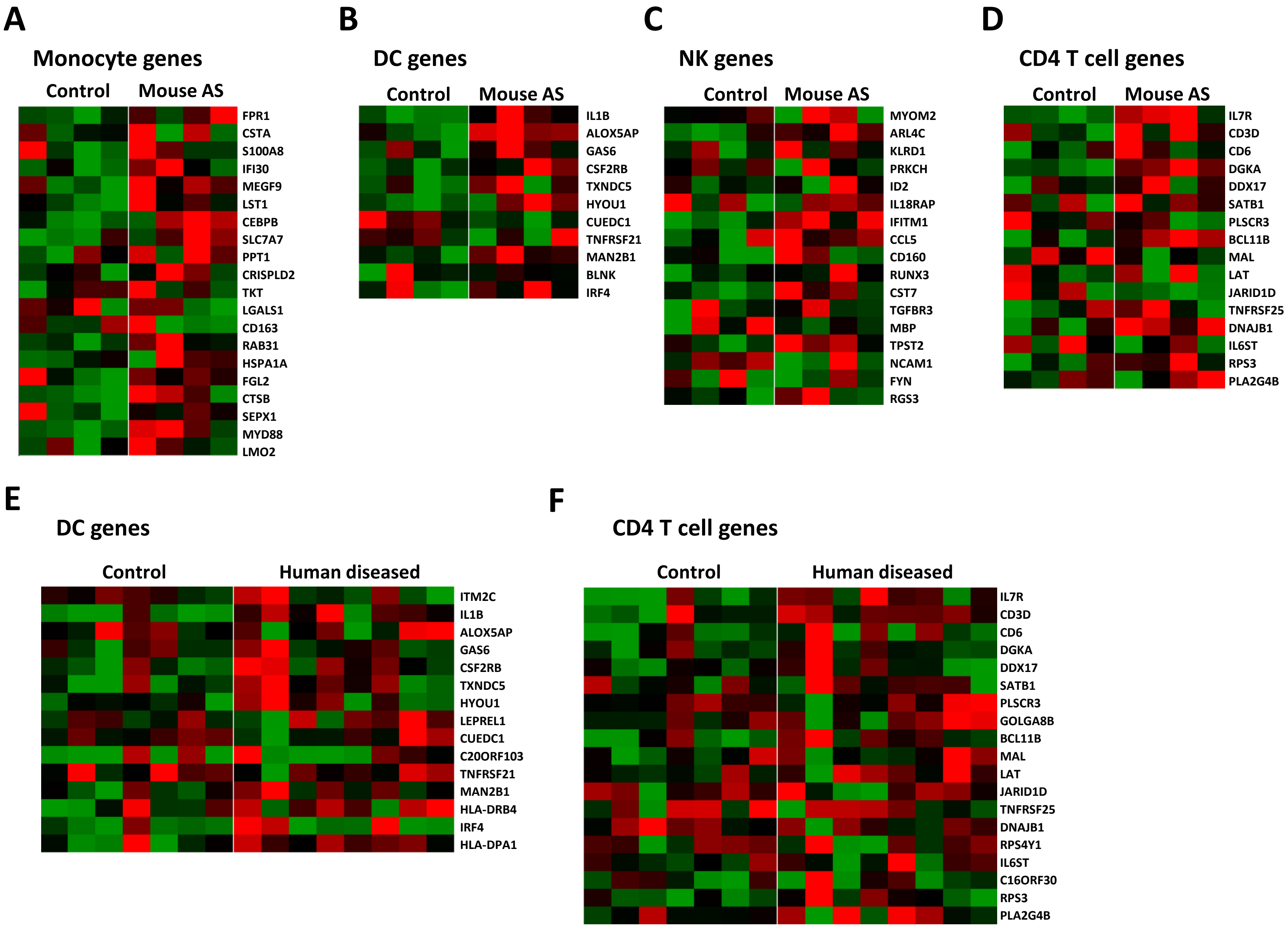

Supplement: Supplementary file 3 — Additional file 3: Figure S2: Gene-centric evaluation of differentially expressed leukocyte metagenes in the mouse (A-D) and human (E & F) datasets. Description of data: Heatmap representation of gene expression in cell types identified in this study. Genes comprising the leukocyte metagene were analyzed for differential expression between healthy and diseases, and indicate no clear pattern of single gene-driven differences in metagene scores. (PDF 61 KB) [file 12891_2014_2396_MOESM3_ESM.pdf]
